# Anti-Ha anti-synthetase syndrome presenting as rapidly progressive interstitial lung disease: a case report of high confidence autoantibody testing

**DOI:** 10.3389/fimmu.2026.1750916

**Published:** 2026-02-27

**Authors:** Alina G. Liedtke, Thomas Daikeler, Stefan Gherca, Matthias J. Herrmann, Spasenija Savic Prince, Sarah L. Tansley, Ingmar A. F. M. Heijnen, Katrin E. Hostettler

**Affiliations:** 1 Clinic of Respiratory Medicine, University Hospital Basel, Basel, Switzerland; 2 Department of Rheumatology, University Hospital of Basel, Basel, Switzerland; 3 Clinic of Radiology and Nuclear Medicine, University Hospital of Basel, Basel, Switzerland; 4 Pathology, Institute of Medical Genetics and Pathology, University Hospital Basel, Basel, Switzerland; 5 Department of Life Sciences, University of Bath, Bath, United Kingdom; 6 Division Medical Immunology, Laboratory Medicine, University Hospital Basel, Basel, Switzerland

**Keywords:** anti-Ha, anti-synthetase syndrome, case report, cyclophosphamide, diagnostic test, interstitial lung disease, myositis, rituximab

## Abstract

We report on a 38-year-old patient who presented with rapidly progressive interstitial lung disease (ILD), without any signs of muscular involvement. Antinuclear antibody testing by indirect immunofluorescence revealed a nuclear titer of 1:320 with a fine speckled and a cytoplasmic titer of 1:1’280 with a fine speckled pattern. Subsequent myositis-specific and myositis-associated antibody tests with commercial multiplex dot-immunoassays showed a strong positive result for anti-Ha antibodies, also confirmed by protein immunoprecipitation, establishing the diagnosis of anti-synthetase syndrome with associated ILD. Despite initial improvement after treatment with intravenous cyclophosphamide and high dose steroids, he relapsed shortly after, with additional muscular symptoms. Subsequent escalation of therapy with rituximab resulted in sustained remission. Considering the scarcity of data about the clinical presentation and prognosis of patients with anti-Ha antibodies, our report provides additional information on diagnostic challenges and therapeutic response in these patients.

## Introduction

Anti-synthetase syndrome (ASyS) is a subset of idiopathic inflammatory myopathies (IIM), characterized by autoantibodies against one of the aminoacyl-transfer RNA (tRNA) synthetases. ASyS is associated with typical clinical features such as interstitial lung disease (ILD), myositis, mechanic’s hands, Raynaud’s phenomenon, and nonerosive arthritis (1–3). Other types of IIM include dermatomyositis, polymyositis, immune-mediated necrotizing myopathy, inclusion body myositis, and overlap myositis, each associated with different autoantibodies (1). Clinical manifestations, treatment response and prognosis are variable, as each type is thought to have different pathophysiological mechanisms (1). An accurate detection of autoantibodies is important for establishing the correct diagnosis and to guide further treatment (1). Whilst testing for the most prevalent anti-synthetase antibody (i.e., anti-Jo1) can be reliably performed by using commercial immunoassays, the detection of less frequent anti-synthetase antibodies (e.g., anti-PL12, anti-PL7, anti-OJ, anti-EJ, anti-Zo, anti-KS, anti-Ha) poses a substantial challenge in daily clinical practice (4). The commercially available assays that are currently in use for these latter non-anti-Jo1 antibodies by clinical laboratories worldwide have variable and limited performance. This may lead to false negative and particularly false positive test results, which can contribute to erroneous diagnoses and treatments (5, 6). To address this problem, an open communication by laboratories has been advocated to ensure that the requesting clinician is aware of the limitations of the performed tests and that results are interpreted in the appropriate manner (7). Furthermore, it is important that laboratories perform high confidence testing of anti-synthetase antibodies by implementing confirmatory tests for patients who have tested positive for rare non-anti-Jo1 anti-synthetase antibodies (8).

With this report, we aim to present a rare case of a patient with rapidly progressive ILD who was found positive for anti-tyrosyl-tRNA synthetase (anti-Ha) antibodies by high confidence antibody testing and responded well to therapy with rituximab.

## Case description

### Presentation and diagnosis

**Figure 1:**
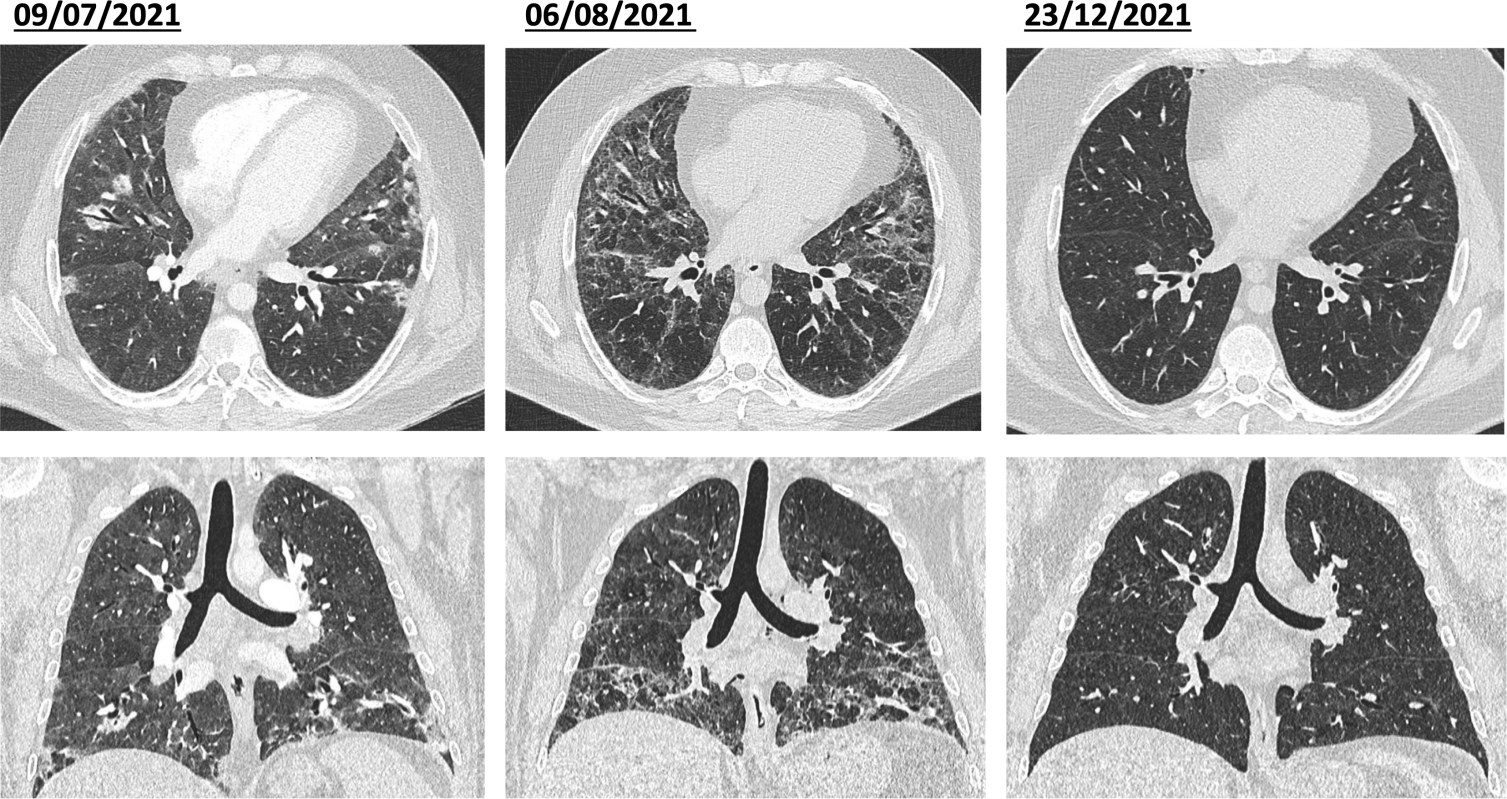
*Initial diagnosis and response to cyclophosphamide and azathioprine.* Left: First presentation with multiple patchy areas of ground-glass opacities with peripheral and peribronchovascular distribution and basal predominance. Middle: Rapidly progressive ILD with extensive ground-glass opacities and interstitial thickening with mild bronchiectasis are seen bilaterally. Right: Near-total resolution of the interstitial changes and bronchiectasis, with minor residual ground-glass opacities remaining after steroid therapy and cyclophosphamide.

A 38-year-old male, Caucasian patient was admitted to our hospital due to worsening dyspnea and dry cough which had been present for approximately three weeks, as well as an unexplained weight loss of 5 kg. He was an active smoker, obese with a BMI of 37 kg/m2, but with no prior significant illness. The clinical examination revealed bibasal fine crackles on lung auscultation and a peripheral oxygen saturation of 90% on ambient air. Chest computer tomography (CT) showed multiple patchy areas of ground-glass opacification in a peripheral and peribronchovascular distribution and with basal predominance (**Figure 1**, left panels). Laboratory tests revealed a leucocytosis of 13.4 x 10^9^/L with left shift and a slightly raised CRP of 16.2 mg/l, while CK was in the normal range.

**Figure 2:**
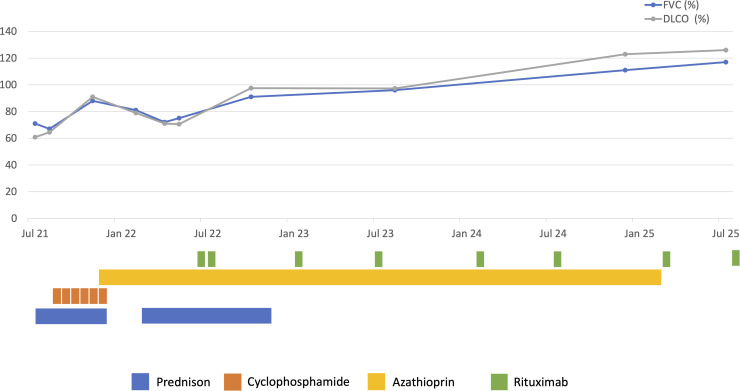
*Pulmonary function tests and medication timeline*. Timeline of pulmonary function parameters (FVC and DLCO) from initial presentation with rapidly progressive ILD, through improvement, relapse, and subsequent stable remission (above). Corresponding immunosuppressive therapies administered at each phase are shown below (Prednisone, Cyclophosphamide, Azathioprine, and Rituximab).

There were no positive findings in the sputum culture or multiplex PCR for respiratory viruses, including SARS-CoV-2 collected by nasopharyngeal swab. Empirical treatment with amoxicillin/clavulanic acid and clarithromycin was initiated upon initial suspicion of atypical pneumonia. Over the following days no clinical improvement was observed. On day five, pulmonary function testing indicated a mild restrictive pattern (forced vital capacity (FVC) 71% predicted, total lung capacity (TLC) 78% predicted) and a decrease in CO lung diffusion capacity (DLCO) of 61% predicted (**Figure 2**). SpO2 was 88% on ambient air.

**Figure 3:**
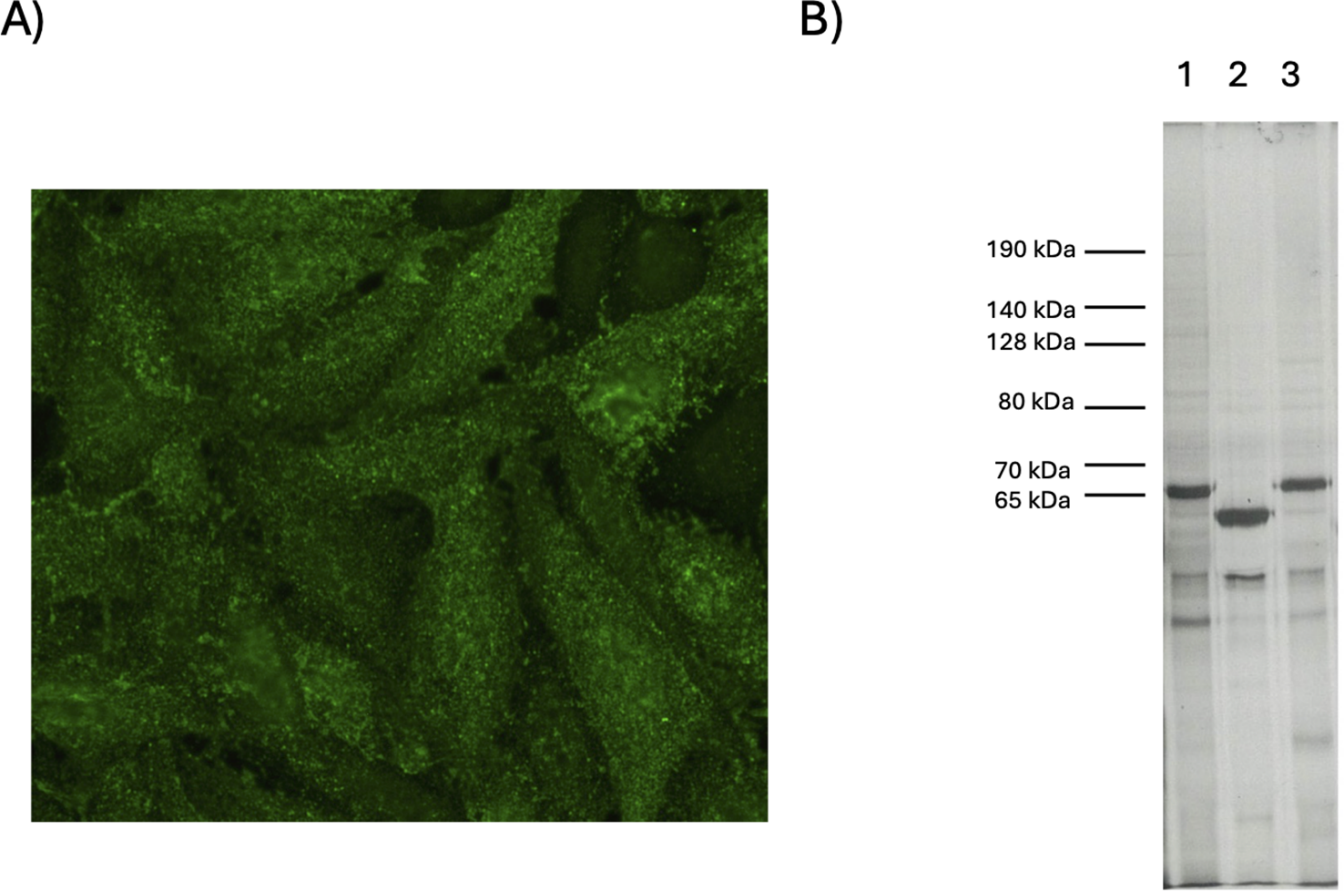
*HEp-2 indirect immunofluorescence (IIF) and immunoprecipitation*. **(A)** HEp-2 IIF, titer 1:160, visualized with NOVA View (Inova Diagnostics) showing a nuclear fine speckled pattern (AC-4 according to ICAP (www.anapatterns.org) and a cytoplasmic fine speckled pattern (AC-20). **(B)** Immunoprecipitation using ^35^S-methionine/cysteine labeled K562 cell extract; lane 1, control patient (anti-KS positive (65 kDa)); lane 2, index patient (anti-Ha positive (58 kDa)); lane 3, control patient (anti-KS positive (65 kDa)). Molecular weights are indicated on the left of panel **(B)**.

The patient underwent bronchoscopy. The bronchoalveolar lavage was not representative for the alveolar compartment. Transbronchial biopsies revealed features of a subacute, focally acute lung injury with organizing pneumonia, focal fibrin exudation, and a mixed interstitial inflammatory infiltrate without fibrosis. Overall, the histologic findings were nonspecific and, after exclusion of infection or drug-induced pneumopathy, compatible with a rheumatic or systemic autoimmune disease. Steroid therapy was initiated (50 mg prednisolone per day). The CRP values further increased up to 38 mg/l, while leucocytosis decreased to 10.8 x 10^9^/L. The patient subsequently insisted on being discharged home (on day eight of hospitalization). Prednisone therapy was maintained at 50 mg/day. Over the following weeks, the patient reported ongoing dyspnea on minor exertion and intermittent coughing. Twenty-six days after discharge, he was again admitted to our hospital. Pulmonary function testing showed a worsening restrictive pattern and gas exchange abnormalities (FVC 67% predicted, DLCO 65% predicted) (**Figure 2**), and a chest CT scan demonstrated a rapid progression of the ILD with extensive ground-glass opacities and interstitial thickening with mild bronchiectasis bilaterally (**Figure 1**, middle panels). A repeat bronchoalveolar lavage uncovered a marked increase in lymphocytes (39%) and a mild increase in neutrophils (14%) without evidence of infectious agents. Meanwhile, antinuclear antibody (ANA) testing by indirect immunofluorescence on HEp-2 cells (Inova Diagnostics, San Diego, CA) revealed a nuclear titer of 1:320 with a fine speckled pattern (AC-4 according to ICAP (www.anapatterns.org), and a cytoplasmic titer of 1:1’280 with a fine speckled pattern (AC-20) (**Figure 3A**). Antibodies against extractable nuclear antigens (ENA) (i.e., Sm, RNP, SSA/Ro60, SSB/La, Scl70) analyzed by fluorescence enzyme immunoassay (FEIA) on the Phadia 250 instrument (Thermo Fisher Scientific, Freiburg, Germany) were negative. Subsequent myositis-specific and myositis-associated antibody tests with commercial multiplex dot-immunoassays (BlueDiver SYNTHE10DBD and MYOS12ADBD kits, D-tek/Alphadia, Mons, Belgium) showed a strong positive result for anti-Ha antibodies (96 arbitrary units (AU); cutoff 10 AU). Other autoantibodies were negative by these dot-immunoassays except for strongly positive anti-Ro52 antibodies (87 AU; cutoff 10 AU), which was confirmed by FEIA on the Phadia 250 system (Thermo Fisher Scientific). Importantly, protein immunoprecipitation, performed as described previously (9), identified a strong band at approximately 58 kDa consistent with anti-Ha (**Figure 3B**). A detailed clinical evaluation was unremarkable, i.e. no signs of arthritis or myositis, normal muscle strength, normal CK level, and no skin abnormalities, particularly no mechanic’s hands and no Raynaud’s phenomenon. Nailfold capillaroscopy was unremarkable. Based on these findings the diagnosis of anti-Ha ASyS with associated ILD was made.

### Initial therapy

Due to progression of ILD despite steroid treatment, the patient received immunosuppressive therapy consisting of cyclophosphamide (15 mg/kg) iv and high-dose methylprednisolone (1’000 mg iv) for 3 days with rapid clinical improvement (SpO2 95% on ambient air, no dyspnea at rest, normalization of CRP). Steroid therapy was maintained with an initial daily dose of 80 mg of prednisolone After discharge, the patient was regularly seen in the outpatient Pulmonology and Rheumatology clinic. After one month, there was a slight improvement in the respiratory function, but dyspnea on exertion persisted (NYHA II-III). Six cycles of cyclophosphamide were administered in total, prednisone was gradually tapered to 20 mg. One and three months post initiation of cyclophosphamide treatment, the patient exhibited further improvement in lung volumes (FVC 88% predicted and TLC 86% predicted after 3 months) and diffusion capacity (DLCO normalization, 92% predicted since first presentation – see **Figure 2**), corresponding to an improvement in dyspnea on exertion and overall performance. CT scan showed near-total resolution of the interstitial changes and bronchiectasis, with minor residual ground-glass opacities remaining (**Figure 1**, right panels). No musculoskeletal symptoms were evident during the clinical assessment at this point after 6 cycles of cyclophosphamide. A maintenance therapy involving azathioprine 150 mg daily was initiated four months after onset of symptoms, and prednisolone was gradually reduced and then completely stopped.

### Relapse

**Figure 4:**
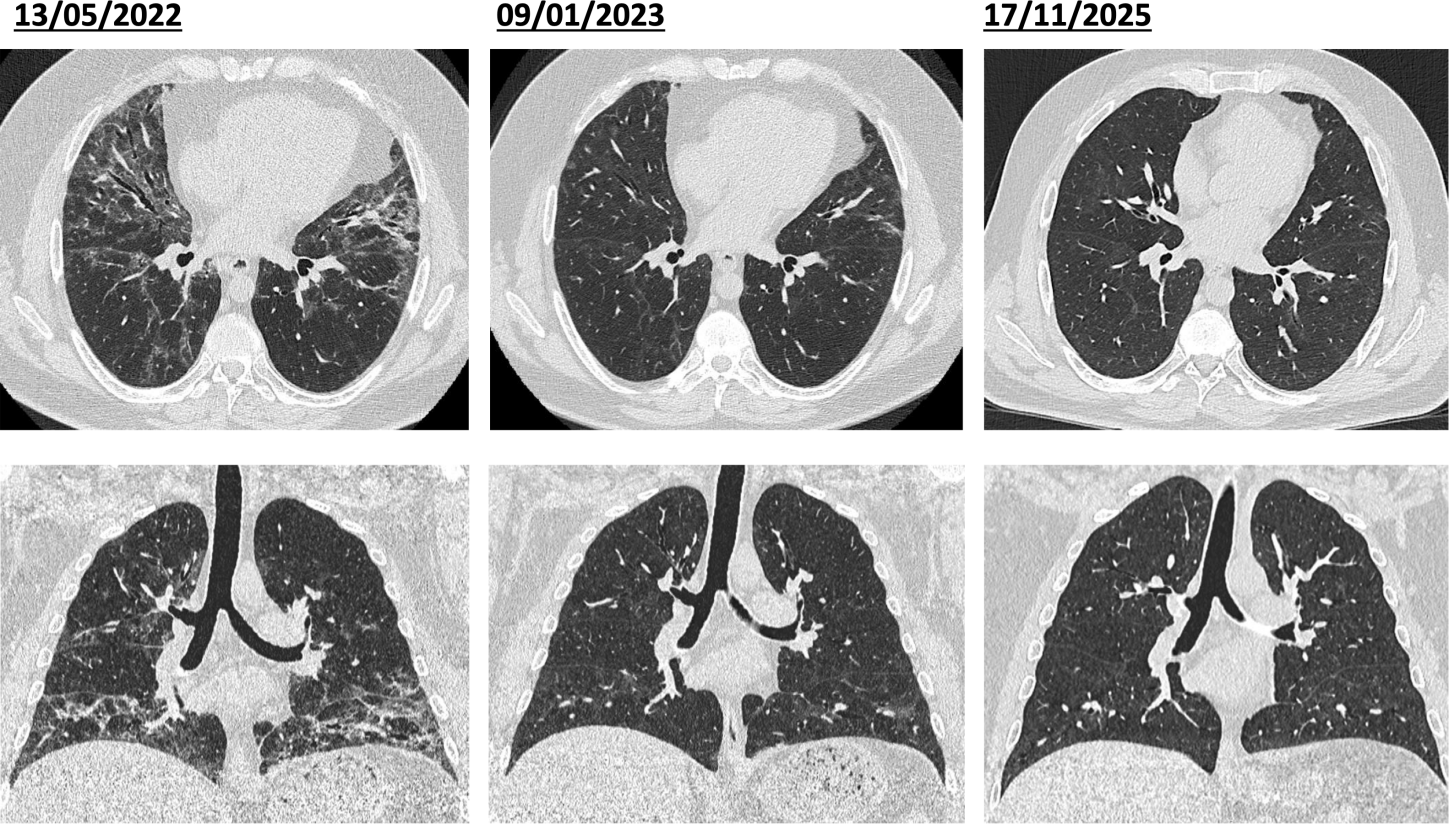
*Relapse and response to rituximab*. Left: Recurrence despite maintenance therapy with Azathioprine with similar changes to the initial CT characterized by extensive reticulation and interstitial thickening, along with ground-glass opacities and bronchiectasis in both lungs, predominantly affecting the basal regions. Middle: Near-total resolution of the ground-glass opacities and bronchiectasis after escalation of therapy to Rituximab. Right: morphological normalization of the lung parenchyma under maintenance monotherapy with Rituximab.

Six months after the initial diagnosis and on treatment with azathioprine 150 mg daily alone the patient reported muscle soreness, subjective weakness and increasing fatigue, with laboratory testing revealing an elevation in CK levels (up to 836 U/L), as well as a modest elevation of ALT and AST (up to 2x ULN) and myoglobin (178 μg/l). Clinical assessment did not detect a decrease in muscle strength, EMG confirmed chronic denervation of the right lateral vastus muscle. The clinical picture was deemed consistent with possible myositis associated with ASyS. Steroid therapy was re-initiated, and azathioprine increased to 200 mg and subsequently to 250 mg daily. This resulted in rapid improvement of muscular symptoms and normalization of CK levels. MRI of the lower limbs performed while the patient was taking 30 mg of prednisone did not show any sign of muscle oedema. However, shortly thereafter, the patient reported worsening of dyspnea on exertion, and PFT showed a considerable decrease in lung volumes (see **Figure 2**). Chest CT scans showed a recurrence with similar changes to the initial CT characterized by extensive reticulation and interstitial thickening, along with ground-glass opacities and bronchiectasis in both lungs, predominantly affecting the basal regions, suggesting a relapse of ASyS-associated ILD. Therapy was thus escalated to rituximab with an initial dosage of 1’000 mg iv, administered twice, 14 days apart. This resulted in an improvement of respiratory symptoms and in PFT values (**Figure 2**). Azathioprine was gradually tapered to a dose of 150 daily. In the subsequent weeks, a clinical, radiological and functional improvement was observed (**Figures 2**, **4**). Rituximab treatment was continued at a dose of 500 mg iv every six months, leading to a sustained steroid-free remission.

### Follow up and outcome

Four years after the initial diagnosis, the patient’s condition remains stable and there have been no further pulmonary or musculoskeletal relapses under rituximab therapy; no adverse events related to rituximab therapy occurred. Nicotine consumption was ceased upon the initial diagnosis. Notably, the patient experienced significant weight gain during high-dose steroid therapy, with BMI increasing from 37 kg/m² to 44.6 kg/m², leading to weight-related comorbidities, including obstructive sleep apnea, prediabetes, and dyslipidemia. Conservative measures, including treatment with a GLP-1 agonist, were unsuccessful. Consequently, bariatric surgery was performed. The patient did not experience immunosuppression-related perioperative complications and achieved a weight loss of 46 kg within one year, also resulting in a significant improvement in lung volumes (**Figure 2**). Of note, a repeat anti-Ha test showed a lower antibody level (65 AU) as compared to the first measurement at the time of diagnosis (96 AU). However, due to the lack of a reliable reference change value for this parameter, it remains unknown whether the detected decrease in anti-Ha levels represents a statistically significant change.

## Discussion

We here report on a rare case of anti-Ha ASyS who initially presented as isolated, rapidly progressive ILD. Patients with ASyS present often with ILD and might have different clinical features, response to treatment and prognosis compared to other IIM (2). Due to lack of physician awareness and clinical research, ASyS may be an under-diagnosed cause of ILD (2). The exact prevalence and incidence of ASyS remain unknown and the current literature is limited.

ASyS is commonly classified as a subset of IIM. However, a substantial proportion of patients do not exhibit myositis. In fact, the prevalence of ILD is higher than myositis (10). Therefore, patients with ASyS may not meet the current ACR/EULAR classification criteria for IIM (10). This was the case for our patient, who had a 2.6 points score (low probability, not meeting the criteria for IIM) (11), while fulfilling the 2010 Connor’s criteria for ASyS (2). Similar to our case, it has been reported that some patients may have a single organ involvement at initial diagnosis but develop other symptoms later in the course of the disease (2, 12). There is thus a consensus on the need for data-based classification criteria specifically for ASyS. International collaborations have recently been initiated to develop a consensus on nomenclature and classification criteria for ASyS (8). Importantly, these initiatives specifically address the different aspects of serological autoantibody testing, including methodology and accuracy. For definite ASyS a high confidence anti-synthetase antibody positive result is required, which - depending on the antibody type - may include the confirmation by a second method (e.g., immunoprecipitation) where a positive result is detected by line- or dot-immunoassay (8).

The first aminoacyl-tRNA synthetase antibody was discovered in the 1980s and was found to target histidyl-tRNA synthetase (Jo1). Anti-Jo1 remains the most frequently detected antibody present in 15-30% of IMM cases (1, 3). The other anti-synthetase antibodies are much less common with anti-PL7 and anti-PL12 each being reported in 5-15% of IMM patients, and the remaining known anti-synthetase antibodies, including anti-EJ, anti-OJ, anti-Zo, anti-KS and anti-Ha, in less than 5% (3).

The presence of anti-Ha antibodies was first reported in an abstract in 2005 (13). Since then, anti-Ha antibodies have been described in limited case reports and case series (14–19). It should however be noted that the case series relied on the use of a single antibody detection method only, such as multiplex line-immunoassay.

Line- and dot-immunoassays to detect anti-synthetase antibodies have become widely available and are offered by a continuously expanding number of clinical laboratories worldwide. Testing is now accessible for clinicians of all disciplines, leading to a significant increase in the number of test requests and a consequent decrease in the prevalence of ASyS in the tested population (i.e., pre-test probability). The assays themselves lack standardization, and as demonstrated for a number of anti-synthetase antibodies, may have limited diagnostic specificity (20). Moreover, the rarity of certain antibodies such as anti-Ha has hampered assay validation, yielding challenges in test result interpretation. A positive test result with limited specificity combined with low pre-test probability generates a low post-test probability of disease and -thus- high probability of a false positive test. In our case, the dot-immunoassay showed a strong positive result for anti-Ha. It has been demonstrated for other anti-synthetase antibodies that a strong positive result has higher specificity as compared to low positive results (21, 22). In addition, the high cytoplasmic titer of the ANA test, which is consistent with the presence of anti-synthetase antibodies, and the detected anti-Ro52 antibodies further increased the likelihood of ASyS in our case as both are significantly associated with its diagnosis (23, 24). Finally, since the positive anti-Ha dot-immunoassay was confirmed by protein immunoprecipitation we can be confident in the diagnosis of an anti-Ha ASyS (8).

Of note, in addition to the current patient, a further three out of 1’368 serum samples (0.2%) tested previously in our laboratory were found positive for anti-Ha antibodies using the same dot-immunoassay. These three cases had a low positive anti-Ha result, and none had a compatible cytoplasmic staining pattern on ANA testing. Two out of the three cases had clinical data available. None of these patients had any clinical feature of ASyS, suggesting false anti-Ha positivity and illustrating the limited performance of this assay when used as a single test.

Little is known about the clinical presentation and prognosis of patients with anti-Ha antibodies. In a small cohort of ILD patients found to be positive for anti-Ha, alternative patterns (other than UIP) were the predominant finding in HRCT (15). Most subjects had preserved pulmonary function, and it was hypothesized that these patients may have a milder disease course (15).

In the absence of randomized controlled trials, there are no evidence-based guidelines for the treatment of ASyS. Treatment recommendations are usually based on ILD secondary to inflammatory myopathies in general (2). First-line treatment usually consists of corticosteroids (e.g., prednisone 1 mg/kg/day, up to 7.5 mg/kg methylprednisolone in severe, life-threatening cases) (1, 2, 25). Given the prolonged course of therapy that is usually required, corticosteroid-sparing agents such as mycophenolate mofetil or azathioprine are often added to induce and maintain remission (2). Cyclophosphamide may be used in severe cases of ILD (1, 2). Due to its systemic toxicity and potential interactions, tacrolimus is often considered a second- or third-line agent (1, 2). In recent years, rituximab for B cell-depletion has emerged as an effective and safe treatment option in refractory ASyS (1, 2, 26, 27). Patients in previous case reports on anti-Ha ASyS were found to have a milder clinical course, responding well to azathioprine (19) or tacrolimus (16, 17) in addition to initial therapy with corticosteroids (16, 17, 19). To the best of our knowledge, this is the first case of anti-Ha ASyS successfully treated with rituximab.

Randomized control trials are needed to determine the best treatment options in this clinically heterogeneous disease.

## Conclusion

We described a case of rapidly progressive ILD associated with ASyS and rare anti-Ha antibodies detected by high confidence antibody testing. While the initial immunosuppressive treatment with cyclophosphamide and oral corticosteroids was successful, the patient relapsed shortly thereafter despite maintenance therapy with azathioprine. Subsequent escalation of therapy with rituximab-induced B cell-depletion resulted in sustained remission and nearly total reversibility of ILD. Considering the scarcity of data about the clinical presentation and prognosis of patients with anti-Ha antibodies, our report provides additional information on diagnostic challenges and therapeutic response in these patients.

## Data Availability

The original contributions presented in the study are included in the article/supplementary material. Further inquiries can be directed to the corresponding author.
